# Mother-reported parental weight talk and adolescent girls’ emotional health, weight control attempts, and disordered eating behaviors

**DOI:** 10.1186/2050-2974-1-45

**Published:** 2013-12-27

**Authors:** Katherine W Bauer, Michaela M Bucchianeri, Dianne Neumark-Sztainer

**Affiliations:** 1 Department of Public Health and Center for Obesity Research and Education, Temple University, 1301 Cecil B. Moore Avenue, Ritter Annex 9th Floor, Philadelphia, PA 19122, USA; 2 Division of Epidemiology and Community Health, School of Public Health, University of Minnesota, 1300 S. 2nd Street, WBOB 3rd floor, Minneapolis, MN 55455, USA

**Keywords:** Parenting, Adolescents, Disordered eating

## Abstract

**Background:**

The aim of this paper is to explore the relationships between mothers’ report of parental weight talk about her daughter, herself, and others, and adolescent girls’ weight-related behaviors and cognitions among a socio-demographically diverse population of mothers and their adolescent daughters.

**Methods:**

Data were drawn from the baseline assessment of 218 mother/adolescent daughter dyads. Mothers completed survey items regarding the frequency of weight talk by parents, and girls completed survey items assessing outcomes including body dissatisfaction, depressive symptomology, use of extreme weight control methods, and binge eating.

**Results:**

More frequent comments to daughters about their weight were associated with higher depressive symptomology (p = 0.041), greater prevalence of extreme weight control behaviors (p = 0.040), and greater prevalence of binge eating (p = 0.048) among girls after adjustment for socio-demographic characteristics and girls’ standardized body mass index (BMI). For example, among girls whose parents never commented on their weight, 4.2% reported use of any extreme weight control behaviors, while 23.2% of girls whose parents frequently commented on their weight reported use of any of these behaviors. Mothers’ more frequent talk about their own weight, shape, or size was associated with lower self-worth (p = 0.007) and higher depressive symptomology (p = 0.004) among girls.

**Conclusions:**

Frequent parental weight talk as perceived by mothers was associated with adolescent girls’ use of harmful weight control methods and poor psychological health, while no associations were found between weight talk and girls’ use of healthful weight control strategies. Interventions that help parents create a family environment that supports healthful activities while reducing weight-related talk may be particularly effective in decreasing the prevalence of harmful outcomes among adolescent girls.

## Background

Among adolescent girls, weight-related problems such as body dissatisfaction, disordered eating, and use of extreme methods to lose weight are highly prevalent, inter-related, and deleterious to girls’ health and well-being [1]. In an effort to identify risk factors for the development of weight-related problems among adolescent girls, several cross-sectional (e.g. [2-7]) and longitudinal (e.g. [8-10]) studies have examined the potential roles of parental behavior and parent–child interactions around weight, body shape and size, and dieting. This body of work has generally suggested that excessive talk about weight by families is correlated with harmful behaviors in youth. Specifically, direct weight talk, or comments directed toward the adolescent regarding their weight and dieting, have been found to be associated with higher levels of depressive symptoms, preoccupation with weight loss, and unhealthy weight control behaviors in cross-sectional studies among adolescent girls [4-7,11]. Some have hypothesized that indirect weight talk, or that for which the child is not the focus, such as a mother’s comments about her own weight, may have less of an association with adolescents’ negative outcomes; however, the body of literature on indirect weight talk is small and has produced inconsistent findings [7,12-14].

Social Cognitive Theory (SCT) [15] provides a basis for understanding the role of parental weight talk in adolescent girls’ health beliefs and behaviors. SCT suggests that environmental and personal factors interact to affect health behaviors. Sociocultural values and norms can be communicated and intensified through behavior modeling by others to influence individuals’ personal beliefs and behaviors. Thus, the pervasive cultural importance placed on slenderness for girls may be further emphasized through mothers’ modeling of weight-related beliefs and behaviors through direct and indirect weight talk [16]. These environmental factors of social norms and parental behavior modeling may then contribute to girls’ personal beliefs about her body and weight, as well as general psycho-social health, which in turn influence her weight-related behaviors.

The majority of studies exploring associations between parental behaviors, including family weight talk, and adolescents’ weight-related problems have utilized data reported by the youth themselves. For example, in a recent review of both cross-sectional and longitudinal studies examining parental correlates of youths’ body image disturbance and disordered eating, 41 of 56 studies included data only reported by adolescents [17]. Studies that assess adolescent-perceived parental behavior have fairly consistently observed associations between parental behavior and adolescent outcomes [12,18,19]. Meanwhile, examinations of parent-reported behavior have yielded mixed results [2,18,19]. Several suggestions have been offered for why adolescents’ report has been more strongly tied to adolescents’ behavior than parents’ report, including that adolescents who are preoccupied with their weight and engaging in unhealthy weight control behaviors perceive their parents’ actions to be about weight or encouraging of dieting even if the parent did not intend for it to be so [20]. Regardless of the reason why adolescents’ perceptions are more predictive of their own behavior, a perception by researchers that parents’ perspective of family weight talk is less important [10] limits identification of potential intervention opportunities with families. With little research utilizing parent report of weight talk, researchers and clinicians are left unsure whether parents’ behavior is a priority target area for intervention, or whether working with the adolescent directly to alter their perceptions of family weight talk may be sufficient to reduce risk.

Given the importance of understanding the role of family weight talk, both direct and indirect, in adolescent girls’ cognitions and behaviors, this study aims to examine the cross-sectional relationships between mother-reported parental weight talk and a wide range of daughters’ outcomes including depression, self-esteem, use of weight control behaviors, and prevalence of binge eating. This study draws upon a socio-demographically diverse sample of high school-aged girls, an important addition to the literature as many previous studies of weight control and disordered eating outcomes have utilized primarily higher socio-economic status, Caucasian study populations [8,21,22], despite the comparable prevalence of unhealthy weight control behaviors and low body satisfaction among adolescents from many racial and ethnic groups [23,24].

## Methods

### Study design

Data for this study were drawn from the baseline assessment of 218 dyads of mothers and their adolescent daughters who participated in New Moves, a school-based intervention designed to prevent obesity and other weight-related problems. Detailed information about the New Moves intervention trial has been published elsewhere [25]. Twelve high schools in the Minneapolis/St Paul metropolitan area participated in the New Moves intervention trial during either the 2007/2008 or 2008/2009 school years. On average across the 12 schools, 56% of students were eligible for free or reduced school breakfast and lunch. Of the 356 girls who participated in New Moves, 71% (253) of their parents completed a survey that assessed characteristics of the family environment. Of the 253 parents who completed a parent survey, 86.2% (218) identified themselves as a mother, stepmother, or other female guardian, and therefore were included in the current study. Data from fathers were not included as there were an insufficient number of fathers in the study to make valid conclusions.

Girls completed baseline data collection during either the end of spring semester or beginning of fall semester preceding their participation in New Moves. The large majority of data collection occurred at the University of Minnesota’s General Clinical Research Center, with a small number of girls completing the study measures at their school. An invitation for parents to participate in the study coupled with a parent survey was mailed to the girls’ homes after parents provided consent for their daughter’s participation in New Moves. The majority of parents completing surveys did so via mail, while 2% of the 253 participating parents completed the survey over the phone with the assistance of trained study staff. The study was approved by the University of Minnesota’s Institutional Review Board and by each participating school district. Parents and girls provided consent/assent for their participation in the study.

### Study sample

Among the 218 mothers/caregivers, 91.3% reported that they were the adolescent girls’ mother, 2.3% reported that they were their stepmother, and 6.4% reported that they were another female guardian. Fairly equal proportions of mothers reported each of the 4 levels of educational attainment ranging from less than a high school degree to completing college. The sample was racially and ethnically diverse with 24.3% of girls reporting they were African-American, 29.8% white, 9.6% Hispanic/Latina, and 24.8% Asian. Approximately half (50.5%) of girls were underweight or normal weight, 17.9% were overweight, and 31.7% were obese. Girls’ were on average 15.6 (SD = 1.1) years old.

### Measures

Items included on the survey completed by girls were tested for overall comprehension and item/scale psychometrics among a sample of girls who participated in a pilot test of the New Moves intervention. To determine reliability of the variables, 48 girls completed the survey twice with two weeks in between. For each variable, the correlation between the responses to each survey was calculated to determine test/retest reliability. An additional test of reliability was performed to determine internal consistency of each scale using data from the sample of girls who participated in the full New Moves intervention trial (n = 356). Psychometric information from these methods for each measure is reported below. Selection of constructs for the survey completed by parents was guided by previous research that identified components of the family environment associated with youths’ weight and weight-related behaviors. Survey items were selected based on their psychometric qualities from previous research, and pilot tested by 10 parents of adolescents for applicability and comprehension.

#### Parental weight talk

The frequency of three types of weight talk: parental weight talk directed at daughters, mothers’ weight talk directed at themselves, and mothers’ weight talk about others, were reported by the mothers utilizing the following three questions, “How often do either you or your spouse/significant other make comments to your daughter about her weight?”; “How often do you talk about your own weight, shape, or size?”; and “How often do you make comments about other people’s weight, shape, or size?” Response options were on a 5-point Likert scale and included: “never,” “rarely,” “sometimes,” “often,” and “very often.” These items were adapted from previously utilized measures of parental weight talk that demonstrated good psychometric properties [26]. Due to the low frequency of mothers reporting use of weight talk directed at their daughters or others “very often,” this category was combined with “often” to create a 4 category variable for the three types of weight talk.

#### Girls’ body dissatisfaction

Girls’ body dissatisfaction was measured with a modified version of the Body Shape Satisfaction Scale [24,27]. Ten questions assessed satisfaction with weight, height and different areas of the body (e.g. stomach, thighs). Questions had six response categories from “very dissatisfied” to “very satisfied” and scores across the 10 questions were summed with higher values representing greater dissatisfaction (test/retest r = 0.84, Cronbach’s α = 0.92).

#### Girls’ self-worth

Self-worth was measured with five questions of perceived global self-worth from the Harter Global Self Worth scale [28]. Girls were asked to compare two statements and identify which one described them better, and then note whether that statement was “Really true for me” or “Sort of true for me.” An example of statements that girls selected from are, “Some teenagers are often disappointed with themselves” and “Other teenagers are pretty pleased with themselves” (test/retest r = 0.78, Cronbach’s α = 0.79).

#### Girls’ depression

Depressive symptoms were measured using the 6-item Kandel and Davies scale [29], which has been validated in an adolescent population. Girls were asked, “During the PAST MONTH, how often have you been bothered or troubled by…” “Feeling too tired to do things”; “having trouble going to sleep or staying asleep”; “feeling unhappy, sad, or depressed”; “feeling hopeless about the future”; “feeling nervous or tense”; and “worrying too much about things.” Response options included “not at all,” “sometimes,” or “very much.” Higher scores are indicative of more depressed mood (test-retest r = 0.83, Cronbach’s α = 0.79).

#### Girls’ weight control behaviors

Weight control behaviors were assessed with the question “Have you done any of the following things in order to lose weight or keep from gaining weight during the past month?”(yes/no). Behaviors categorized as extreme unhealthy weight control behaviors included: 1) took diet pills, 2) made myself vomit, 3) used laxatives, and 4) used diuretics (test/retest r = 0.70). Unhealthy weight control behaviors included all of the above and also: 1) fasted, 2) ate very little, 3) used food substitutes, 4) skipped meals, 5) smoked more cigarettes, and 6) gone on a diet (test/retest r = 0.80). Healthy weight control behaviors included: 1) exercised, 2) ate more fruits and vegetables, 3) ate fewer high fat foods, 4) drank less regular soda pop or sweetened beverages, 5) paid attention to portion sizes, and 6) ate fewer sweets (test/retest r = 0.83) [24]. Mean levels of the total number of healthy weight control behaviors reported and the percent of girls engaging in any unhealthy weight control behaviors and extreme weight control behaviors were calculated.

#### Girls’ binge eating

Binge eating with loss of control was assessed with two questions. Girls were first asked, “In the past month, have you ever eaten so much food in a short period of time that you would be embarrassed if others saw you (binge eating)?” If girls responded yes to this question, they were asked to respond to the question: “During the time when you ate this way, did you feel you couldn’t stop eating or control what or how much you were eating?” Response options were “yes” or “no.” Girls were classified as engaging in binge eating with loss of control if they answered “yes” to both questions (test/retest r = 1.00) [30,31].

#### Covariates

Girls self-reported their own race/ethnicity and girls’ ages were calculated from their birth date as recorded on their consent form. Girls’ body mass index (BMI) was calculated from girls’ weight and height as measured by trained study staff. BMI was calculated using the formula: weight in kilograms divided by height in meters squared and was converted to age-adjusted z-scores and percentiles, and girls’ weight status was determined with girls with a BMI percentile less than 5 categorized as underweight, less than 85 categorized as normal weight, between 85 and 95 categorized as overweight, and greater than or equal to 95 categorized as obese [32,33]. Only 2.3% of girls in the sample were underweight, so the underweight and normal weight categories were combined for analysis.

### Statistical analysis

Frequency of socio-demographic characteristics of mother/daughter dyads, percent of daughters who were underweight/normal weight, overweight, and obese, and frequency of the three types of weight talk were calculated. To examine associations between mother-reported weight talk and the girls’ outcomes represented continuously (i.e., body dissatisfaction, self-worth, depression, and number of healthy weight control behaviors used), hierarchical linear regression models were developed with separate models for each of the three types of weight talk. Girls’ race/ethnicity and age were included as covariates. Additionally, girls’ BMI z-score was included as a covariate in order to remove confounding of the relationship between weight talk and girls’ outcomes by girls’ relative weight. A variable representing the school that girls attended was included in the models as a random effect to account for correlation of outcomes among girls who attended school together. Adjusted means and associated standard errors were generated, and a test of linear trend of the mean level of girls’ outcomes across the 4 levels of weight talk was conducted. For the dichotomous outcomes of unhealthy weight control behaviors, extreme weight control behaviors, and binge eating with loss of control, hierarchal logistic regression models including girls’ race/ethnicity, age, and BMI z-score as covariates and a random effect for girls’ school were developed. The predicted probabilities for each observation in the data set at the observed value of the covariate variables were computed, and the average of these predicted values (which can be viewed as a generalization of standardization to the total study population) was reported. A test of linear trend of the average predicted probabilities across the 4 levels of weight talk was conducted.

## Results

Twenty-seven percent of mothers reported commenting on their own weight, shape, or size often or very often, while a smaller proportion reported that either they or their significant other commented on their daughters’ weight often or very often, or reported they commented on others’ weight, shape, or size often or very often (12.4% and 13.0%, respectively). Nearly one third (30.9%) of mothers reported that they or their significant other never comment on their daughter’s weight (Table 1). Moderate correlations ranging from 0.31 to 0.39 were observed between the 3 types of weight talk (data not shown).

**Table 1:** Frequency of mother-reported weight talk among participants in the New Moves study

	**% (n)**			
	**Never**	**Rarely**	**Sometimes**	**Often/very often**
**Parent comments to daughter about her weight**	30.9 (67)	28.1 (61)	28.6 (62)	12.4 (27)
**Mother talks about own weight/shape/size**	9.2 (20)	22.6 (49)	41.0 (89)	27.2 (59)
**Mother comments on others’ weight/shape/size**	25.0 (54)	31.5 (68)	30.6 (66)	13.0 (28)

Parental weight talk, as reported by mothers, was moderately associated with less healthful cognitions or behaviors among girls after adjustment for socio-demographic characteristics and girls’ BMI z-score (Table 2). Greater frequency of parents’ comments to their daughter regarding her weight, a measure of direct weight talk, was associated with higher levels of girls’ depression (p = 0.041), a higher prevalence of using at least one extreme weight control behavior (p = 0.040), and a higher prevalence of binge eating with loss of control (p = 0.048). For example, within families where comments were never made to girls about their weight, only 4.2% of the girls utilized extreme weight control methods, while within families where comments were made about the girls’ bodies often or very often, 23.2% of the girls utilized extreme weight control methods. Similarly, 27.5% of girls whose parents commented on their weight often or very often reported binge eating, while only 8.1% of girls whose parents never commented on their weight binge ate. Mothers’ frequency of talk about her own weight, shape, or size was associated with girls’ report of lower self-worth (p = 0.007) and higher depressive symptomology (p = 0.004), while mothers’ comments about others’ weight, shape, or size were associated with higher depressive symptomology (p = 0.047). Of the three types of weight talk assessed, none were associated with a higher use of healthy weight control behaviors.

**Table 2:** Associations between mothers’ weight talk and adolescent girls’ body dissatisfaction, self-worth, depression, and weight control and disordered eating behaviors*

	**Body dissatisfaction**	**Self-worth**	**Depression**	**Healthy weight control behaviors**	**Unhealthy weight control behaviors**	**Extreme weight control behaviors**	**Binge eating with loss of control**
	**(Range 10–60)**	**(Range 5–20)**	**(Range 6–18)**				
	**Mean (SE)**	**Mean (SE)**	**Mean (SE)**	**Mean (SE)**	**%**	**%**	**%**
**Parent comments to daughter about her weight**							
Never	33.7 (1.6)	15.1 (0.5)	11.1 (0.4)	3.5 (0.2)	65.3	4.2	8.1
Rarely	31.8 (1.6)	14.4 (0.5)	11.6 (0.4)	4.0 (0.2)	60.1	9.2	13.7
Sometimes	35.8 (1.6)	14.7 (0.5)	11.0 (0.4)	3.6 (0.2)	70.4	6.4	13.6
Often/very often	38.4 (2.5)	13.8 (0.7)	12.9 (0.6)	3.6 (0.4)	81.4	23.2	27.5
P (trend)	0.052	0.213	0.041	0.986	0.127	0.040	0.048
**Mother talks about own weight/shape/size**							
Never	34.6 (2.7)	16.5 (0.8)	9.9 (0.6)	4.1 (0.4)	72.4	13.5	5.0
Rarely	33.8 (1.8)	15.1 (0.5)	11.5 (0.4)	3.1 (0.3)	56.7	3.2	8.4
Sometimes	33.3 (1.3)	14.6 (0.4)	11.2 (0.3)	3.8 (0.2)	64.0	6.2	11.6
Often/very often	36.2 (1.6)	14.0 (0.5)	12.2 (0.4)	3.6 (0.2)	78.1	14.1	23.0
P (trend)	0.649	0.007	0.004	0.597	0.529	0.728	0.095
**Mother comments on others’ weight/shape/size**							
Never	37.4 (1.6)	15.5 (0.5)	11.1 (0.4)	3.5 (0.3)	68.5	4.8	11.6
Rarely	33.0 (1.5)	14.2 (0.4)	11.2 (0.4)	3.8 (0.2)	62.2	8.6	9.3
Sometimes	32.0 (1.5)	14.9 (0.4)	11.4 (0.4)	3.6 (0.2)	63.0	8.4	13.4
Often/very often	36.7 (2.3)	14.0 (0.7)	12.4 (0.6)	3.7 (0.4)	84.3	14.1	27.3
P (trend)	0.718	0.155	0.047	0.873	0.155	0.162	0.061

*Models adjusted for girls’ race/ethnicity, age, and BMI z-score.

## Discussion

Among a socio-economically and racially/ethnically diverse sample of adolescent girls, mothers commonly reported that they discussed their own weight, shape, or size at least sometimes. Weight talk directed at daughters and talk about others’ weight, shape, and size were less common than mothers’ talk about her own weight, shape, or size, although still prevalent. Higher levels of all three types of parental weight talk were correlated with greater depressive symptomology among girls. Weight talk directed at the girl was more consistently associated with negative outcomes than weight talk about the mothers themselves or others. None of the types of weight talk were associated with greater use of healthful weight control behaviors among girls. This finding aligns with previous research that observed that mothers’ dieting behaviors and maternal encouragement of daughters to diet were not correlated with greater use of healthful weight control strategies among daughters [5]. Together these results are important as some mothers may believe that weight comments directed toward daughters or themselves will prompt greater use of healthful weight control strategies among their daughters; however, study results do not provide evidence that this is the case.

Findings from the present study extend those of Smolak, et al. [7] and Haines, et al. [14] that utilized parental report of weight comments to examine negative outcomes among elementary school-aged samples of children. In these studies, mothers’ comments regarding their child’s weight were positively associated with the child’s poor body esteem, concern about gaining weight, and attempts to lose weight. Similarly in the current study, mothers’ or their significant others’ comments to their daughter about her weight were associated with depressive symptoms and use of extreme weight control behaviors such as taking diet pills or vomiting to lose weight. These findings remained after adjustment for girls’ BMI z-score, strengthening the evidence that engaging in talk about adolescent girls’ weight is associated with weight-related problems independent of girls’ objective weight. Further, findings from the current study highlight the potential harm of mothers’ weight talk directed toward herself and weight talk about others, behaviors that are quite normative in our society. These types of indirect weight talk were associated with girls’ reports of lower self-worth and higher depressive symptomology.

As compared to the study by Neumark-Sztainer, et al. [12], which examined associations between girls’ report of mothers’ weight talk and weight and eating-related outcomes among girls participating in the New Moves intervention, fewer significant relationships between mothers’ behavior and girls’ outcomes were observed in the current study. Together the findings from these two studies utilizing the New Moves population suggest that adolescents may be perceiving their parents’ messages differently than they are intended. However, mothers may also report their participation in weight talk less accurately than their children due to a social desirability bias, or may not be aware of the extent that they and their spouse/significant other comment on their daughter’s weight, or mothers comment on their own or others’ weight. Regardless, findings of this and other studies suggest that both mothers’ perception of parental behavior and daughters’ perception of their mothers’ behavior may be risk factors for depression, use of extreme weight control behaviors, and binge eating among adolescent girls. Therefore, weight talk in the home as perceived by both parents and children may need to be addressed in efforts to prevent disordered eating and other weight-related problems among adolescent girls.

This study has several strengths and limitations. The socio-demographic diversity of the sample is a study strength, particularly as many previous studies examining mother-daughter relationships have be conducted with more homogeneous populations [8,21]. Additionally, the assessment of multiple types of weight talk allowed for an examination of the prevalence of these behaviors and their associations with girls’ outcomes. Further, a wide range of girls’ outcomes was measured including those specific to weight control and disordered eating, and general psychological outcomes including depressive symptomology and self-worth. Limitations of the study include that the measure of direct weight talk asked mothers about their own as well as their spouses’/significant others’ comments toward their daughters. This measure was constructed in such a way to elicit less biased responses from mothers, who may feel hesitant to report the full extent of their comments toward their daughters, but limits our understanding of the role of weight talk from mothers specifically. Another limitation is that additional measures of mothers, such as their BMI or depressive symptomology, were not assessed in this study. These unmeasured variables may in part explain the observed associations between parental weight talk and daughters’ outcomes. Further, as data for the study were cross-sectional, temporality of the relationships between weight talk and girls’ outcomes cannot be determined. Therefore, it is not known whether girls who exhibit high degrees of weight and body concern elicit more talk about weight by their parents.

## Conclusions

Among the high school-aged girls who participated in the New Moves study, parental weight talk as reported by mothers was associated with harmful weight control methods and poor psychological health among adolescent girls. These results support the need for further research regarding the content, context, and role of weight talk among a wider variety of population groups. Assessment of the frequency and types of weight talk among, multiple family members would contribute to a fuller understanding of weight talk perceptions among, and dynamics between, parents and children. Further, future studies should explore objective methods to assess weight talk, such as through observations of family meals or general interactions. Triangulation of objective and subjective information would aid in identifying behavioral targets to reduce the occurrence or perception of weight talk. Additional research is specifically needed to understand fathers’ use of weight talk and the influence of weight talk to adolescent boys, as few studies have included boys, despite the relatively high prevalence of unhealthful weight control and disordered eating among this population [34]. In our society, discussion of weight and body size is ubiquitous, and adolescents are frequently exposed to messages about body weight and the importance of thinness through the media, peers, and their families. Because of this pervasiveness of messaging, parents may not be aware that excessive parental focus on their and others’ weight may be harmful to their children’s health. Further, parents may believe that making comments about their child’s and their own weight can motivate positive actions and engagement in healthy behaviors, although there is no evidence to support these beliefs. Recently several studies have identified parenting practices that are associated with a healthy weight and a lower prevalence of unhealthy weight control practices among adolescents including parents’ modeling of healthful eating, having nutritious foods accessible in the home, providing frequent family meals, and talking with children about positive behaviors, such as eating nutritious foods, as opposed to dieting and weight loss [35-37]. Therefore, interventions that work with parents to increase their knowledge and skills regarding these positive parenting practices, and reduce weight-related talk and teasing directed toward self, children, and others may be particularly effective in decreasing the prevalence of disordered eating, dangerous weight control behaviors, body dissatisfaction, and depressive symptomology among adolescents.

## Abbreviations

BMI: Body mass index.

## Competing interests

The authors declare that they have no competing interests.

## Authors’ contributions

KWB: Made substantial contributions to conception and design, or acquisition of data, and analysis and interpretation of data; was involved in drafting the manuscript; and gave final approval of the version to be published. MMB: Made substantial contributions to conception and design and analysis and interpretation of data; was involved in critically revising the manuscript; and gave final approval of the version to be published. DNS: Made substantial contributions to conception and design and analysis and interpretation of data; was involved in critically revising the manuscript; and gave final approval of the version to be published.

## References

[B1] Neumark-Sztainer D Story M Hannan PJ Perry CL Irving LM Weight-related concerns and behaviors among overweight and nonoverweight adolescents: implications for preventing weight-related disorders Arch Pediatr Adolesc Med 2002 1 171 178 10.1001/archpedi.156.2.171 11814380

[B2] Cooley E Toray T Wang MC Valdez NN Maternal effects on daughters’ eating pathology and body image Eat Behav 2008 1 52 61 10.1016/j.eatbeh.2007.03.001 18167323

[B3] Meesters C Muris P Hoefnagels C van Gemert M Social and family correlates of eating problems and muscle preoccupation in young adolescents Eat Behav 2007 1 83 90 10.1016/j.eatbeh.2006.02.002 17174855

[B4] Hanna AC Bond MJ Relationships between family conflict, perceived maternal verbal messages, and daughters’ disturbed eating symptomatology Appetite 2006 1 205 211 10.1016/j.appet.2006.02.013 16701919

[B5] Fulkerson JA McGuire MT Neumark-Sztainer D Story M French SA Perry CL Weight-related attitudes and behaviors of adolescent boys and girls who are encouraged to diet by their mothers Int J Obes Relat Metab Disord 2002 1 1579 1587 10.1038/sj.ijo.0802157 12461674

[B6] Armstrong B Janicke DM Differentiating the effects of maternal and peer encouragement to diet on child weight control attitudes and behaviors Appetite 2012 1 723 729 10.1016/j.appet.2012.06.022 22885728

[B7] Smolak L Levine MP Schermer F Parental input and weight concerns among elementary school children Int J Eat Disord 1999 1 263 271 10.1002/(SICI)1098-108X(199904)25:3<263::AID-EAT3>3.0.CO;2-V 10191990

[B8] Francis LA Birch LL Maternal influences on daughters’ restrained eating behavior Health Psychol 2005 1 548 554 16287400 10.1037/0278-6133.24.6.548 PMC2562308

[B9] Westerberg-Jacobson J Edlund B Ghaderi A Risk and protective factors for disturbed eating: a 7-year longitudinal study of eating attitudes and psychological factors in adolescent girls and their parents Eat Weight Disord 2010 1 e208 e218 21406944 10.1007/BF03325302

[B10] van den Berg PA Keery H Eisenberg M Neumark-Sztainer D Maternal and adolescent report of mothers’ weight-related concerns and behaviors: longitudinal associations with adolescent body dissatisfaction and weight control practices J Pediatr Psychol 2010 1 1093 1102 10.1093/jpepsy/jsq042 20498008 PMC2980944

[B11] Schreiber GB Robins M Striegel-Moore R Obarzanek E Morrison JA Wright DJ Weight modification efforts reported by black and white preadolescent girls: national heart, lung, and blood institute growth and health study Pediatrics 1996 1 63 70 8668414

[B12] Neumark-Sztainer D Bauer KW Friend S Hannan PJ Story M Berge JM Family weight talk and dieting: how much do they matter for body dissatisfaction and disordered eating behaviors in adolescent girls? J Adolesc Health 2010 1 270 276 10.1016/j.jadohealth.2010.02.001 20708566 PMC2921129

[B13] Rodgers RF Paxton SJ Chabrol H Effects of parental comments on body dissatisfaction and eating disturbance in young adults: a sociocultural model Body image 2009 1 171 177 10.1016/j.bodyim.2009.04.004 19464242

[B14] Haines J Neumark-Sztainer D Hannan P Robinson-O’Brien R Child versus parent report of parental influences on children’s weight-related attitudes and behaviors J Pediatr Psychol 2008 1 783 788 10.1093/jpepsy/jsn016 18304997 PMC2734118

[B15] Bandura A Vasta R Social cognitive theory Annals of child development Vol 6 Six theories of child development. Volume 6 1989 Greenwich, CT: JAI Press 1 60

[B16] Pike KM Rodin J Mothers, daughters, and disordered eating J Abnorm Psychol 1991 1 198 204 2040771 10.1037//0021-843x.100.2.198

[B17] Rodgers R Chabrol H Parental attitudes, body image disturbance and disordered eating amongst adolescents and young adults: a review Eur Eat Disord Rev 2009 1 137 151 10.1002/erv.907 19130467

[B18] Keel PK Heatherton TF Harnden JL Hornig CD Mothers, fathers, and daughters: dieting and disordered eating Eat Disord 1997 1 216 228 10.1080/10640269708249227

[B19] Baker CW Whisman MA Brownell KD Studying intergenerational transmission of eating attitudes and behaviors: methodological and conceptual questions Health Psychol 2000 1 376 381 10907656 10.1037//0278-6133.19.4.376

[B20] Keery H Eisenberg ME Boutelle K Neumark-Sztainer D Story M Relationships between maternal and adolescent weight-related behaviors and concerns: the role of perception J Psychosom Res 2006 1 105 111 10.1016/j.jpsychores.2006.01.011 16813852

[B21] Field AE Austin SB Striegel-Moore R Taylor CB ACC Jr Laird N Colditz G Weight concerns and weight control behaviors of adolescents and their mothers Arch Pediatr Adolesc Med 2005 1 1121 1126 10.1001/archpedi.159.12.1121 16330734

[B22] Field AE Javaras KM Aneja P Kitos N Camargo CA Jr Taylor CB Laird NM Family, peer, and media predictors of becoming eating disordered Arch Pediatr Adolesc Med 2008 1 574 579 10.1001/archpedi.162.6.574 18524749 PMC3652375

[B23] Deleel ML Hughes TL Miller JA Hipwell A Theodore LA Prevalence of eating disturbance and body image dissatisfaction in young girls: an examination of the variance across racial and socioeconomic groups Psychol Sch 2009 1 767 775 10.1002/pits.20415 20336184 PMC2844708

[B24] Neumark-Sztainer D Croll J Story M Hannan PJ French SA Perry C Ethnic/racial differences in weight-related concerns and behaviors among adolescent girls and boys: findings from Project EAT J Psychosom Res 2002 1 963 974 10.1016/S0022-3999(02)00486-5 12445586

[B25] Neumark-Sztainer DR Friend SE Flattum CF Hannan PJ Story MT Bauer KW Feldman SB Petrich CA New moves-preventing weight-related problems in adolescent girls: a group-randomized study Am J Prev Med 2010 1 421 432 10.1016/j.amepre.2010.07.017 20965379 PMC2978965

[B26] Neumark-Sztainer D Haines J Robinson-O’Brien R Hannan PJ Robins M Morris B Petrich CA Ready. Set. ACTION! A theater-based obesity prevention program for children: a feasibility study Health Educ Res 2009 1 407 420 10.1093/her/cyn036 18622011 PMC2682640

[B27] Pingitore R Spring B Garfield D Gender differences in body satisfaction Obes Res 1997 1 402 409 10.1002/j.1550-8528.1997.tb00662.x 9385613

[B28] Harter S Manual for the self-perception profile for adolescents 1988 Denver, CO: University of Denver

[B29] Kandel DB Davies M Epidemiology of depressive mood in adolescents: an empirical study Arch Gen Psychiatry 1982 1 1205 1212 10.1001/archpsyc.1982.04290100065011 7125850

[B30] Yanovski SZ Nelson JE Dubbert BK Spitzer RL Association of binge eating disorder and psychiatric comorbidity in obese subjects Am J Psychiatry 1993 1 1472 1479 8379549 10.1176/ajp.150.10.1472

[B31] Neumark-Sztainer D Wall M Story M Fulkerson JA Are family meal patterns associated with disordered eating behaviors among adolescents? J Adolesc Health 2004 1 350 359 15488428 10.1016/j.jadohealth.2004.01.004

[B32] Kuczmarski RJ Ogden CL Guo SS Grummer-Strawn LM Flegal KM Mei Z Wei R Curtin LR Roche AF Johnson CL 2000 CDC Growth Charts for the United States: methods and development Vital Health Stat 2002 1 1 190 12043359

[B33] Barlow SE Expert committee recommendations regarding the prevention, assessment, and treatment of child and adolescent overweight and obesity: summary report Pediatrics 2007 1 Suppl 4 S164 S192 18055651 10.1542/peds.2007-2329C

[B34] Neumark-Sztainer D Wall MM Larson N Story M Fulkerson JA Eisenberg ME Hannan PJ Secular trends in weight status and weight-related attitudes and behaviors in adolescents from 1999 to 2010 Prev Med 2012 1 77 81 10.1016/j.ypmed.2011.10.003 22024221 PMC3266744

[B35] Bauer KW Berge JM Neumark-Sztainer D The importance of families to adolescents’ physical activity and dietary intake Adolesc Med State Art Rev 2011 1 601 613 xiii 22423466

[B36] Savage JS Fisher JO Birch LL Parental influence on eating behavior: conception to adolescence J Law Med Ethics 2007 1 22 34 10.1111/j.1748-720X.2007.00111.x 17341215 PMC2531152

[B37] Patrick H Nicklas TA A review of family and social determinants of children’s eating patterns and diet quality J Am Coll Nutr 2005 1 83 92 10.1080/07315724.2005.10719448 15798074

